# A novel design strategy for nanoparticles on nanopatterns: interferometric lithographic patterning of Mms6 biotemplated magnetic nanoparticles†
†Electronic supplementary information (ESI) available. See DOI: 10.1039/c5tc03895b



**DOI:** 10.1039/c5tc03895b

**Published:** 2016-01-12

**Authors:** S. M. Bird, O. El-Zubir, A. E. Rawlings, G. J. Leggett, S. S. Staniland

**Affiliations:** a University of Sheffield , Department of Chemistry , Dainton Building , Sheffield , S3 7HF , UK . Email: s.s.staniland@sheffield.ac.uk; b University of Newcastle , Chemical Nanoscience Laboratories , School of Chemistry , Bedson Building , Newcastle Upon Tyne , NE1 7RU , UK

## Abstract

Nanotechnology demands the synthesis of highly precise, functional materials, tailored for specific applications. One such example is bit patterned media. These high-density magnetic data-storage materials require specific and uniform magnetic nanoparticles (MNPs) to be patterned over large areas (cm^2^ range) in exact nanoscale arrays. However, the realisation of such materials for nanotechnology applications depends upon reproducible fabrication methods that are both precise and environmentally-friendly, for cost-effective scale-up. A potentially ideal biological fabrication methodology is biomineralisation. This is the formation of inorganic minerals within organisms, and is known to be highly controlled down to the nanoscale whilst being carried out under ambient conditions. The magnetotactic bacterium *Magnetospirillum magneticum* AMB-1 uses a suite of dedicated biomineralisation proteins to control the formation of magnetite MNPs within their cell. One of these proteins, Mms6, has been shown to control formation of magnetite MNPs *in vitro*. We have previously used Mms6 on micro-contact printed (μCP) patterned self-assembled monolayer (SAM) surfaces to control the formation and location of MNPs in microscale arrays, offering a bioinspired and green-route to fabrication. However, μCP cannot produce patterns reliably with nanoscale dimensions, and most alternative nanofabrication techniques are slow and expensive. Interferometric lithography (IL) uses the interference of laser light to produce nanostructures over large areas *via* a simple process implemented under ambient conditions. Here we combine the bottom-up biomediated approach with a top down IL methodology to produce arrays of uniform magnetite MNPs (86 ± 21 nm) with a period of 357 nm. This shows a potentially revolutionary strategy for the production of magnetic arrays with nanoscale precision in a process with low environmental impact, which could be scaled readily to facilitate large-scale production of nanopatterned surface materials for technological applications.

## Abstract 2

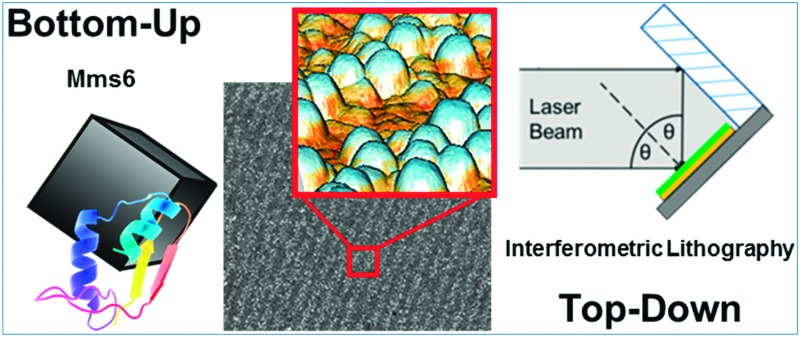
Top-down surface patterning technique, interferometric lithography, is combined with bottom-up magnetite nanoparticle biomineralisation using Mms6 to form magnetic nanoscale arrays.

## Introduction

The advancement of nanotechnology is driven by the ability to fabricate tailored functional materials with nanoscale precision. Magnetic nanoparticles (MNPs) are increasingly found in a number of commercial applications, and therefore development of new synthesis methods with the ability to control the size, shape and crystallinity of MNPs is critical.^1–3^ The precise patterning of MNPs onto surfaces could form a new route to bit-patterned media (BPM), potentially extending the storage capacities of magnetic hard disk drives (HDDs) to form the basis of a new generation of ultra-high density data storage devices.^4,5^

Currently, data is stored within a magnetic HDD by writing information onto a granular ferromagnetic film.^4^ The grains of this film are magnetically oriented to form bits of information, which can be read as binary code. Today, magnetic HDDs have storage capacities in excess of 500 Gbit in^–2^, 20 million times more storage capacity than the first drive introduced in 1956.^4^ This has in the most part been achieved by scaling the components of magnetic HDDs to ever smaller dimensions. However, this trend cannot continue indefinitely. As the demand for data storage continues to grow, current magnetic data storage technology is reaching its physical limit as decreasing MNP size result in enhanced thermal demagnetisation effects and superparamagnetism.^3^

BPM is a new technology able to overcome this physical limitation, which has the promise to dramatically increase data storage-density, forming devices with capacities in the Tbit in^–2^ range.^4–6^ In this case a surface patterned with discrete magnetic “nanoislands” is used, and each bit of information is written onto each individual magnetic nanoisland.^5^ One of the principal challenges to overcome, before BPM becomes a viable storage technology, is the development of an economical method of forming and nanopatterning on a surface the billions of highly uniform magnetic nanoislands that are required.^4^

In this work we have developed a bioinspired and green strategy for the nanoscale fabrication of a MNP array. Precisely controlling the array dimensions along with the crystallisation of the MNPs, without the use of expensive equipment, facilities and processes requirements. The control of the location and properties of the MNPs on the surfaces is achieved with the use of biomineralisation proteins.

In Nature, proteins perform complex synthetic functions. Dedicated biomineralisation proteins produce inorganic mineral structures within biological organisms. Biomineralisation proteins have evolved over millions of years to control the formation of a variety of minerals under mild aqueous conditions.^7^ Many other biomineralising biomolecules have been identified or modified to form precise materials *in vitro*, and to template the formation of abiotic materials (including: gold,^8^ silver,^9^ FePt,^10^ and CoPt^11,12,50^).

Magnetotactic bacteria can form highly uniform MNPs composed of magnetite (magnetic iron oxide, Fe_3_O_4_) within unique lipid organelles termed magnetosomes.^13–16^ The crystallisation of the magnetite MNP is regulated by biomineralisation proteins that are located within the magnetosome membrane.^17,18^ Several proteins were found tightly bound to the MNPs of magnetite in the magnetotactic bacterium *Magnetospirillum magneticum* AMB-1 by Arakaki *et al.* ^18^ One protein in particular, Mms6, contains a hydrophobic N-terminal region for integration into the magnetosome membrane, and an acidic C-terminal region that can strongly bind iron ions and is thought to nucleate and control the formation of magnetite *in vivo*.^18,19^ It has also been shown that purified Mms6 is able to control the formation of MNPs of magnetite *in vitro*.^18–21^

We have used Mms6 previously to biotemplate the formation of MNPs of magnetite onto gold surfaces.^22,23^ Mms6 was patterned onto functionalised gold surfaces through the use of micro-contact printing (μCP). During a magnetite mineralisation reaction Mms6 facilitates both the formation and immobilisation of MNPs on the patterned surface.^22,23^ More recently, we published an adaptation to this approach in which Mms6 was engineered to contain an N-terminal cysteine.^24^ An anti-biofouling oligo(ethylene glycol) terminated (OEG-thiolate) self-assembled monolayer (SAM) was printed onto a gold surface with a flexible polymer stamp, after which the remaining space was backfilled with the cysteine-modified Mms6. This allowed the protein to be immobilised directly onto a gold surface and biotemplated MNP arrays of magnetite and magnetically harder cobalt-doped magnetite were successfully generated.^24^ Furthermore, this route to control the location of Mms6 on the surface did not reduce its biotemplating function.^24^

μCP with traditional Sylgard PDMS stamps is a cheap and simple route to forming patterns of SAMs on surfaces with feature size >500 nm (as only the initial masters need to be produced in a cleanroom).^25^ However, this micron scale patterning is far from the nanoscale precision required for BPM, and achieving patterning consistency across wide areas with μCP is problematic.^25^ Therefore, for biotemplated BPM to become a reality, an alternative approach to patterning is required. For example, patterns of biotemplated materials have been formed with the use of fluidics^9^ and holographic patterning.^26^ Techniques such as electron-beam lithography (EBL),^27^ focussed ion beam (FIB)^28^ and scanning probe techniques such as dip-pen nanolithography (DPN)^29,30^ and nanoshaving^31^ have been shown to achieve patterning resolutions required for BPM. However, these expensive and slow serial patterning techniques are unlikely to ever be scaled up for the mass production of affordable magnetic HDDs.

SAMs can also be modified and patterned by exposure to UV light. Alkylthiolate SAMs are photo-oxidised on exposure to light with a wavelength of 244 nm, converting the strongly bound alkylthiolate to a weakly bound alkylsulfonate that may be displaced by a contrasting adsorbate in a simple solution-phase exchange process.^32,33^ At the nanometer scale, patterns with features as small as 9 nm have been formed using near-field techniques. However, an alternative approach is provided by interferometric lithography (IL),^34^ in which two coherent beams of light are caused to interfere to create an interferogram with sinusoidal cross-section and a period of *λ*/2*n* sin *θ* over the sample surface. Such approaches have been used to pattern SAMs.^35,36^ In regions of the monolayer exposed to a maximum in the interferogram, the adsorbates are photo-oxidised, while in regions exposed to minima, the extent of oxidation is minimal. This approach has enabled dimensions as small as 30 nm to be achieved under ambient conditions, and over wide areas (cm^2^ and above).^36,37^

Here, for the first time, we combine this powerful top-down (IL) nanopatterning with the bottom-up biomineralisation protein Mms6 to create uniform MNPs of magnetite in precise nanoscale patterns. This novel and green approach is a significant step towards addressing the challenge of developing a surface suitable for BPM, and could be adapted to produce a the vast range of new tailored nanoscale surfaces for future devices.

## Experimental

### Synthesis of MNP arrays

#### Synthesis of recombinant cysteine-tagged Mms6 (cys-Mms6)

Synthesis of cysteine tagged Mms6 was performed according to Bird *et al.* ^24^ A summary of the key properties of the protein can be found in ESI,† Fig. S1.

#### Preparation of gold surfaces

Gold surfaces were evaporated onto clean glass microscope slides. These slides were sonicated sequentially for 5 minutes in: 1% Decon 90, ultrapure water, methanol, and ultrapure water. The slides were then dried with nitrogen gas, before being cleaned in a piranha solution (H_2_SO 70% : H_2_O_2_ 30% v/v) for 10 minutes, followed by rinsing with ultrapure water and finally dried with nitrogen. A 5 nm adhesion layer of chromium was applied, and then 50 nm of gold was evaporated onto the slides in an Edwards Auto 360 thermal evaporator. The slides were then scribed and split to form ≈1 cm^2^ substrates.

#### Interference lithography (IL)

An anti-biofouling SAM was formed on the clean gold substrates by immersion in an 1 mM alkanethiol solution (11-mercaptoundecyl tetra(ethylene) glycol (OEG-thiolate), Sigma) in ethanol for 24 hours. The surfaces were then removed from this solution, rinsed in ethanol and dried with nitrogen. The interference lithographic (IL) process was adapted from the method described in Tizazu *et al.* ^37^ IL was carried out by exposing the OEG-thiolate coated surfaces to a Coherent Innova 300C FreD frequency-doubled argon ion laser beam (*λ* ≈ 244 nm, maximum power 100 mW) in a Lloyd's mirror arrangement. The laser beam was expanded so that it illuminated an area of *ca.* 1 cm^2^, and was directed towards the surface fixed at an angle 2*θ* to a mirror. The laser beam was positioned so that half of the beam interacted directly with the sample surface, while the other half reflected off the mirror onto the sample. The power of the laser at the sample surfaces was recorded before the exposure, so that the surfaces were subjected to an optimal dose of 20 J cm^–2^, resulting in spatially defined photodegradation of the SAM layer. The surfaces were then rinsed in ethanol and dried with nitrogen.

#### Attachment of cys-Mms6

The surfaces patterned by IL were immediately placed into a PBS solution at pH 7.4 containing the cys-Mms6 protein (10 μg mL^–1^) for 1 hour. This allowed cys-Mms6 to bind to areas of the gold surface where the OEG-thiolate SAM had be photodegraded, thus selectively functionalising these areas for biomineralisation.

#### Magnetite mineralisation

The protein patterned substrates were then placed into a partial oxidation of ferrous hydroxide with potassium hydroxide (POFHK) reaction,^38^ which was used in previous work to form MNPs of magnetite on biotemplating surfaces.^22–24^ Firstly, the substrates were rinsed in ultrapure water, before being transferred to a glass vessel containing 24.75 mL of anaerobic ultrapure water (vacuum degassed for 1 hour and sparged with nitrogen for 1 hour to remove oxygen before use). Reactants were dissolved into anaerobic ultrapure water to form stock solutions of 0.5 M FeSO_4_·7H_2_O, 1 M KOH and 0.5 M KNO_3_. 2.5 mL of the FeSO_4_ solution and 2.75 mL of the KOH solution were added to the vessel before 20 mL of the KNO_3_ solution was added dropwise over ≈5 minutes. The vessel was then subject to heating at 80 °C for 4 hours in an inert environment. Once complete, the samples were removed from the vessel, rinsed in anaerobic ultrapure water, and dried with nitrogen. The excess magnetite particles that formed in the reaction solution were collected magnetically and washed in ultrapure water five times.

### Characterisation

#### Scanning electron microscopy (SEM)

Samples were fixed onto aluminium stubs with double sided carbon tape and earthed with silver paint. SEM images were recorded on an FEI Inspect F50 SEM at an accelerating voltage of 5–10 keV, a working distance of approximately 10 mm, and processed with xT software.

#### Transmission electron microscopy (TEM)

The MNPs that formed in solution during the POFHK reaction were collected magnetically and dispersed in anaerobic ultrapure water. 10 μL of this suspension was pipetted onto carbon coated copper TEM grids (S162-3, Agar), and the grids were allowed to dry in air. Micrographs were recorded with an FEI Tecnai G2 Spirit TEM operating at 80 keV and processed with Gatan DigitalMicrograph software.

#### Grain size analysis

The grain size of the nanoparticles viewed with SEM and TEM was recorded along the longest axes of the projection using ImageJ software.^39^ ≈100 particles per sample were measured, and these data were compiled into a histogram and fitted with a Gaussian distribution in GraphPad Prism software.‡
‡GraphPad Prism, version 6.01, Graphpad Software Inc., San Diego, CA, USA, 2013.


#### X-ray diffraction (XRD)

Biomineralised MNP surfaces were analysed with XRD using a Siemens D5000 diffractometer in reflection mode. X-rays were generated at 40 kV and 40 mA using a Cu Kα source (average *λ* = 1.54178 Å). X-rays were directed onto surfaces that were mounted on non-elastic Apiezon Q Sealing Compound putty in glancing angle geometry. X-ray intensities were then collected between 2*θ* = 15° and 70° with a position sensitive detector (in 0.025° steps and 2.5 seconds per step).

MNPs that formed from the bulk solution during mineralisation reactions were dried and mixed with Elmer's glue onto acetate disks, and loaded into a STOE STADI P diffractometer. X-rays were generated at 40 keV and 35 mA using a Cu Kα1 source, with X-ray intensities collected between 2*θ* = 15° and 70° (in 0.03° steps and 2.5 seconds per step). Data analysis was performed with Diffrac.Plus TOPAS software, and compared to *d*-spacings in the JCPDS crystallographic database.^40^

The grain size of the MNPs analysed with XRD was calculated with the use of the Debye–Scherrer equation.^41^ This analysis was performed on the 311 peak for each sample, and a shape constant of 0.89 was used.

#### Friction force microscopy (FFM)

Clean gold surfaces were immersed in a 1 mM mixed thiol solution in ethanol containing 90% OEG-thiolate and 10% hexaethylene spaced carboxylic acid terminated alkanethiol (Sigma). After exposure the mixed SAM samples to different doses of laser through the interferometer, samples were immersed in a 2 mM solution of 1-octadecanethiol (Sigma) for 2 hours, rinsed in ethanol and dried with nitrogen. The samples were imaged by FFM. Friction force microscopy images were acquired in air using a Bruker MultiMode 8 NanoScope V AFM. The probes used for FFM were silicon nitride probes (NP series, Bruker) with spring constant *k* = 0.12 N m^–1^.

#### Magnetic force microscopy (MFM)

Atomic force microscopy (AFM) topographical images were recorded on a Multimode Nanoscope III AFM operating in tapping mode with a magnetised Cr/Co coated MESP tip (Bruker). Magnetic perturbations between the magnetised tip and the sample were measured by retracing the topography at a lift height of 50 nm, and recording the phase shift of the cantilever's resonant frequency. This shift is proportional to the strength of magnetic attraction (negative phase shift) or repulsion (positive phase shift) between the particles and the magnetised tip. These data was processed with WSxM software,^42^ and the 3D images were rendered in “R” using the rgl package.§
§The program used to render the MFM images in 3D is available here: https://github.com/jonbramble/MFMPlot.


## Results and discussion

**Fig. 1:**
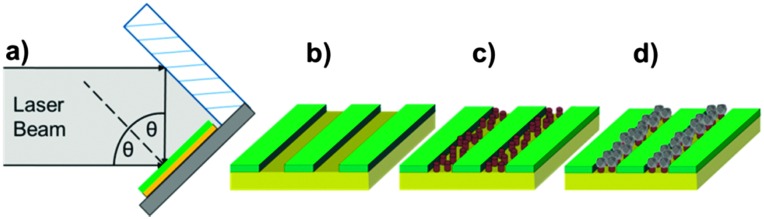
Schematic representing the stages involved in producing the MNP arrays. (a) A gold surface with a complete OEG-thiolate SAM (green) is exposed to laser light in a Lloyd's mirror configuration. (b) Nanopattern formed in the SAM layer. (c) The remaining clean gold space is backfilled with cys-Mms6 (brown cylinders). (d) Selective formation of MNPs (grey crystal) on the protein covered areas after immersion in a POFHK reaction designed to produce magnetite.

Previously, we have shown that cysteine-tagged Mms6 (cys-Mms6) forms almost a complete monolayer on a gold surface, with significantly reduced binding to an antibiofouling OEG-thiolate SAM.^24^ Therefore, the patterning of a OEG-thiolate SAM layer onto gold surfaces forms a route to controlling the location of Mms6 on the surface. Here, a gold surface, covered in a complete OEG-thiolate SAM layer, was exposed to laser light in a Lloyd's mirror interferometer. This led to spatially selective photo-oxidation in regions exposed to a maximum in the interferogram (formed by constructive interference), while minimal modification of the surface occurred in regions exposed to minima in the interferogram (corresponding to destructive interference). The result is the formation of a periodic array of uniformly aligned bands occupied by the OEG-thiolate SAM, separated by regions in which the adsorbate has been photo-oxidised. The photo-oxidised adsorbates are susceptible to displacement from the surface, either by a contrasting adsorbate or, as here, by solvent rinsing to expose the underlying gold surface. The protein cys-Mms6 was adsorbed onto the gold regions formed between the bands of intact OEG-thiolate adsorbates. The patterned surfaces were then subjected to a partial oxidation of ferrous hydroxide with potassium hydroxide (POFHK) reaction to form MNPs of magnetite. A schematic illustration of this process is outlined in Fig. 1.

To determine the optimum exposure in the lithographic process, gold surfaces covered in a mixed SAM of OEG-thiolate and carboxylic acid terminated thiols were exposed for a range of different times, and hence doses. After exposure, the surfaces were backfilled with a CH_3_ terminated thiol, and characterised by friction force microscopy (FFM). The CH_3_ terminated SAM provides good contrast in FFM, because it exhibits a much lower coefficient of friction than the polar adsorbates,^43^ allowing the pattern generated to be readily observed (ESI,† Fig. S2). It was found that an exposure of *ca.* 20 J cm^–2^ was sufficient to create clear features with well-defined contrast in the OEG-thiolate in the SAM, and this dose was selected for the subsequent cys-Mms6 experiments.

**Fig. 2:**
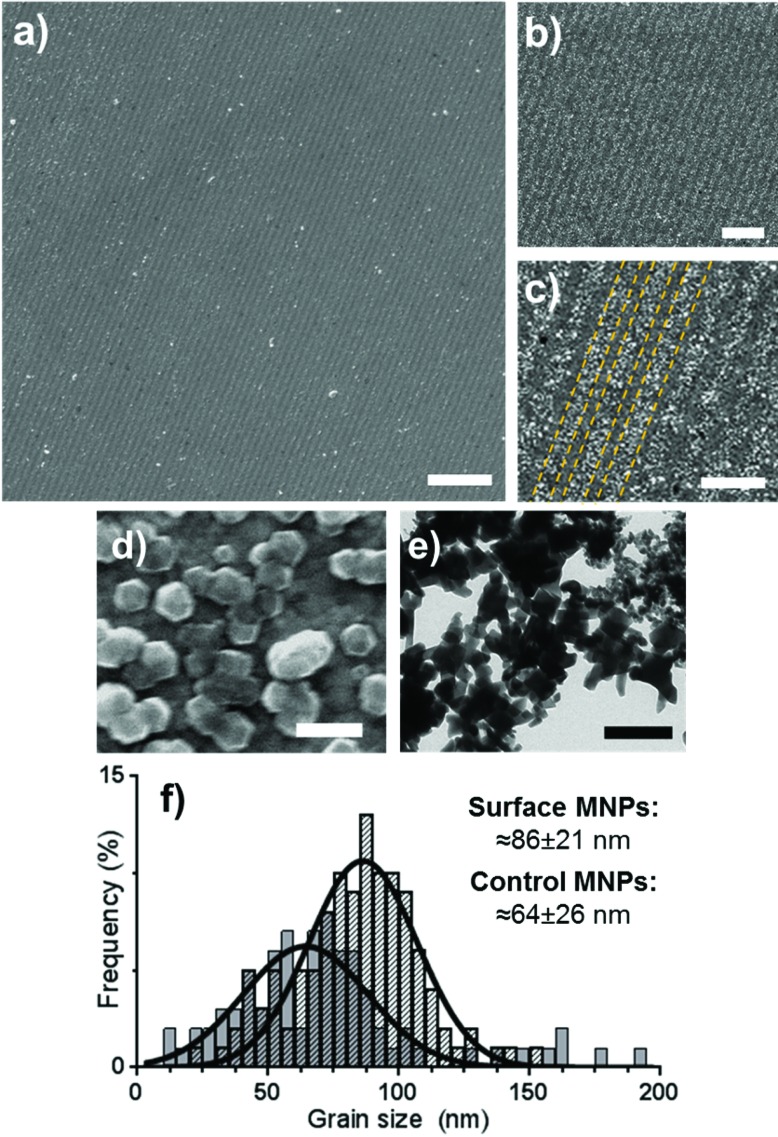
SEM images (a–d) of Mms6 surfaces patterned by IL after a POFHK reaction at different magnifications (yellow dotted lines on image c indicate regions of Mms6 protein and OEG-thiolate SAM). TEM image (e) of MNPs formed in a control POFHK reaction. Scale bars: a – 2 μm, b – 1 μm, c – 500 nm, d – 100 nm and e – 200 nm. Grain size analysis (f) based on ≈100 MNPs per sample. The longest axis of the MNP projections in TEM and SEM images was measured using ImageJ, and results were plotted and fitted with a Gaussian distribution in GraphPad Prism software.‡

Gold surfaces covered with a complete OEG-thiolate SAM were exposed in IL at an angle of 2*θ* = 20°. The surfaces were then backfilled with cys-Mms6 and subjected to a POFHK reaction to form magnetite MNPs on the protein patterns. The resultant surfaces were investigated by scanning electron microscopy (SEM) (Fig. 2). This revealed the presence of line arrays of nanoparticles (corresponding to the protein patterned regions) and regions with negligible mineralisation (corresponding to the OEG-thiolate patterned regions) on the surface (Fig. 2a), with the average period of the pattern measuring 316 nm. We have previously shown that MNPs do not bind to surfaces which are protected by a OEG-thiolate SAM.^24^ This was also the case for the surfaces in this study, where MNPs have formed with high density on the protein patterned areas with only limited binding to the OEG-thiolate SAM regions (Fig. 2).

The MNPs that were formed on the gold surfaces (Fig. 2d) were compared to MNPs produced in a control POFHK reaction (without the addition of any patterned surfaces or protein) (Fig. 2e). Grain size analysis (Fig. 2f) of these two nanoparticle populations shows that the MNPs present on the surface formed with a larger mean size (≈86 ± 21 nm) and smaller size distribution than the control MNPs (≈64 ± 26 nm). This approximately 35% size increase is consistent with our previous studies of surface immobilised Mms6 mediated MNP formation, and shows the protein is actively controlling the MNP crystallisation.^24^ It is believed that the acidic C-terminal region of an assembly of Mms6 on the surface accumulates iron ions, nucleating and controlling the formation of magnetite MNPs. It is noteworthy that Mms6 controls the formation with respect to the size of particles depending on whether the Mms6 is in solution (MNP ≈ 20 nm)^20^ or on a surface (MNP ≈ 86 nm), and this is proposed to be an effect of the curvature of the protein's assembly motif.^44^

**Fig. 3:**
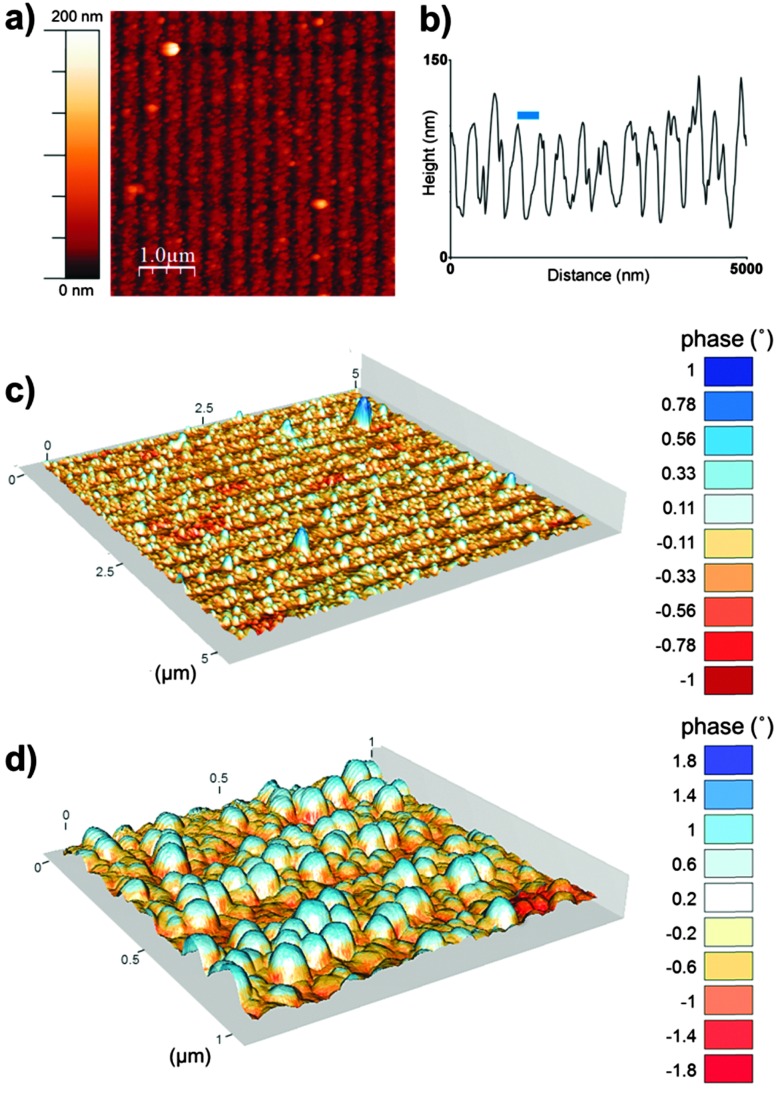
Tapping mode AFM image (a), an example height profile across a section of the tapping mode AFM image (b) and composite images of tapping mode AFM and MFM phase shift at a lift height of 50 nm (c and d, scales in μm) of a MNP array biotemplated by Mms6 after patterning by IL. Blue bar represents the average period (357 nm).

To further characterise the nanoparticle arrays atomic force microscopy (AFM) and magnetic force microscopy (MFM) was performed. Fig. 3 shows a tapping mode AFM image and MFM plots of the MNP arrays. These images also help to show the clarity and uniformity of patterning achieved. As we expected from our SEM analysis the AFM shows a regular line array consisting of a layer of MNPs and regions with negligible MNP formation. The height profile across the tapping mode AFM image (Fig. 3b) defines the average period of the line pattern of MNPs more clearly, and was measured to be 357 nm. This includes lines of biotemplated MNPs with an average width of 274 nm, and a OEG-thiolate SAM background spacing region with an average width of 83 nm. This period can be adjusted by varying the angle *θ* during the IL exposure.^37^ The difference in height between the peak minima and maxima in the height profile gives a thickness of the nanoparticle layer of approximately 90 nm. This is consistent with a single layer of nanoparticles being immobilised on the surface, as the grain size analysis of MNP showed the average particle diameter was 86 nm.

The composite AFM and MFM plots also show zones of attraction and repulsion (red and blue areas respectively). Previously we have shown in MFM studies that zones of attraction extend over multiple Mms6 biotemplated MNPs, and that these zones are stable at room temperature.^22–24^ These previous data and the MFM analysis displayed in Fig. 3 suggest that the MNPs biotemplated by Mms6 are ferrimagnetic.

**Fig. 4:**
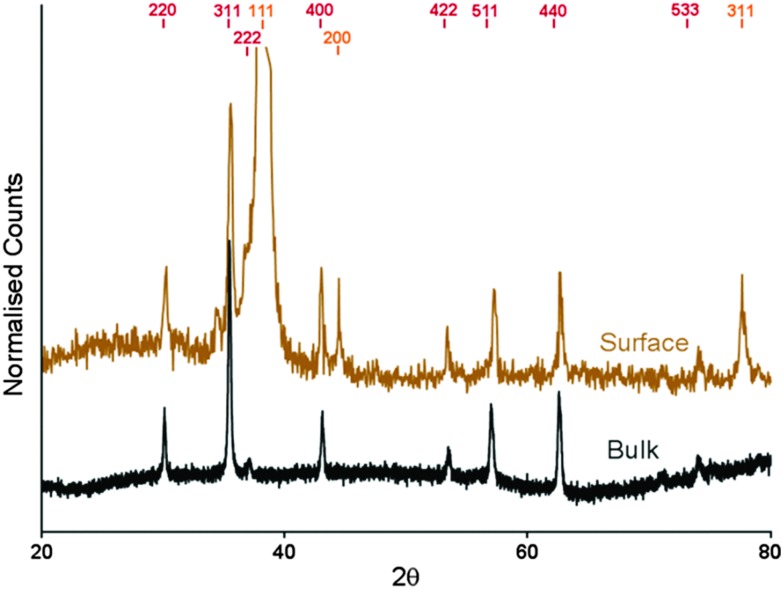
XRD data recorded for the MNPs biomineralised by Mms6 onto gold (gold) and of the control particles that form in solution (black) during a POFHK reaction. The expected peak positions for magnetite (red) and gold (gold) are highlighted.

To confirm that the particles that had formed on the surface were magnetite, we conducted crystallographic analysis of both the MNP patterned surfaces and the control particles that formed in a POFHK reaction using XRD (Fig. 4). The interplanar distances (*d*-spacings) were extrapolated from the position of the diffraction peaks (Table 1). We compared these values to those corresponding to magnetite, and the closely related iron oxide maghemite (available from the JCPDS crystallographic database). For the particles that formed in solution during the POFHK reaction (black data, Fig. 4) the XRD diagram shows peaks at 2*θ* = 30.09°, 35.34°, 37.10°, 43.10°, 53.40°, 56.80°, 62.51° and 73.50°. Similarly, for the MNPs biomineralised onto the gold surface the XRD data (gold data, Fig. 4) shows peaks at 2*θ* = 30.15°, 35.45°, 42.95°, 53.40°, 57.20°, 62.65° and 74.05°. The majority of these peaks were all a good fit to the magnetite (220), (311), (400), (422), (511), (440) and (533) peaks respectively, and a closer fit than the peaks for maghemite (Table 1). The additional peaks at 2*θ* = 38.25°, 44.45° and 77.65° correspond to the Au(111), (200) and (311) reflections from the gold, with the Au(111) peak obscuring the (222) peak for magnetite. However, this analysis provides strong evidence that magnetite was the majority product formed in the control POFHK reaction and biotemplated onto the gold surfaces by Mms6. The (400) plane in particular, which can be used to distinguish between magnetite and maghemite, confirms the majority of the material is most likely to be magnetite.^45^

**Table 1:** Summary of the *d*-spacings for maghemite, magnetite, the control MNPs formed in a POFHK reaction and the MNPs biotemplated on to the gold surface shown in Fig. 4 (all measured in Å)^*a*^

Peak	Magnetite	Maghemite	POFHK_(Bulk)_	Mms6_(surface)_
(220)	2.966	2.950	2.970	2.964
(311)	2.530	2.520	2.540	2.532
(222)	2.419	2.410	2.423	—^*b*^
(400)	2.096	2.080	2.099	2.106
(422)	1.712	1.700	1.716	1.716
(511)	1.614	1.610	1.621	1.610
(440)	1.483	1.480	1.486	1.483
(533)	1.279	1.270	1.288	1.280

^*a*^Maghemite values are from JCPDS card 00-039-1346 and magnetite from card 00-019-0629.

^*b*^Obscured by the Au(111) peak.

The (311) peak was fitted to the Debye–Scherrer equation, to determine the grain size of the MNPs that were biomineralised onto the surfaces and the control MNPs that formed in solution during the POFHK reaction.^41^ This fitting suggested that the control nanoparticles that formed in a POFHK reaction had a mean size of ≈72 nm, while the MNPs biomineralised onto the gold surfaces by Mms6 had a mean size of ≈89 nm. These values confirm the general trend that MNPs were biomineralised onto the gold surfaces by Mms6 with a larger mean size than those that form in solution during a POFHK reaction. However, discrepancies with the mean size calculated from the grain size analysis (Fig. 2) could be a result of the Debye–Scherrer equation, which assumes the particles have a narrow size distribution and are perfectly crystalline.^46^ The fact that the biotemplated surface particles are in closer agreement than the control particles could also be factor of their tighter size distribution.

For the first time, with the use of IL, Mms6 has been used to produce uniform lines of magnetite MNPs with nanoscale precision. This proof of principle experiment demonstrates that nanostructured arrays of magnetite nanoparticles can be biotemplated. Clearly, future work will be needed to address the geometry of the patterns formed, and optimise these for specific applications such as BPM. However, previous work has shown that a very wide range of packing geometries and particle morphologies is readily accessible by the IL patterning of SAMs.^35^

IL can be used to generate dot arrays with nanoscale precision in SAMs, through the application of two identical exposures at 90° angles.^37^ However, we cannot apply this approach to the scheme outlined in Fig. 1 to generate dot arrays of Mms6. In that case, a complete OEG-thiolate SAM would be exposed twice (at 90° angles) to form islands of OEG-thiolate SAM surrounded by areas of unmodified gold. As the OEG-thiolate SAM blocks the attachment of the cys-Mms6 protein this would lead to the majority of the surface being covered by Mms6, the opposite configuration to what is required. In an attempt to address this issue we repeated our experiment to see if we could use IL to selectively remove cys-Mms6 from a surface and backfill with a OEG-thiolate SAM.

**Fig. 5:**
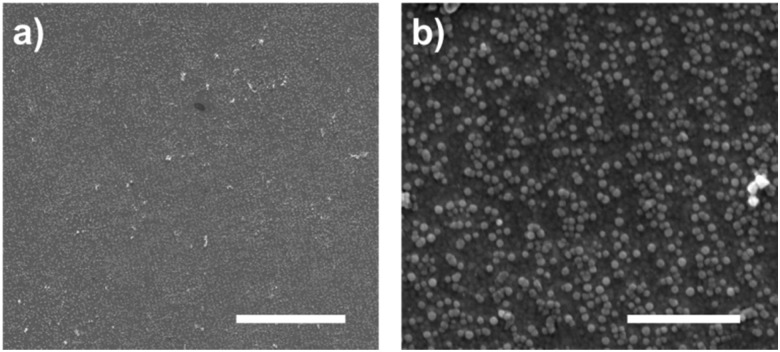
SEM images of gold surfaces covered with a complete layer of cysteine-tagged Mms6 protein that were patterned by IL at an exposure dose of 100 J cm^–2^, after backfilling with OEG-thiolate and being subjected to a POFHK reaction. Scale bars: a – 10 μm and b – 1 μm.

We used clean gold surfaces and immersed them in PBS buffer containing cys-Mms6 so that a complete layer of Mms6 formed. These surfaces were then subjected to exactly the same process as the gold surfaces coated in a OEG-thiolate SAM had been (as shown in Fig. 1). We anticipated that when exposed to the bright fringes the cys-Mms6 on the surface would be degraded. After this treatment, the surface was backfilled with a OEG-thiolate SAM, before being subjected to a POFHK reaction. In this case, the OEG-thiolate SAM does not define the location of the protein on the gold surface, but is still required to block the attachment of MNPs onto the unmodified gold areas during the mineralisation reaction. SEM images of the Mms6 biotemplated MNP arrays formed using this approach are shown in Fig. 5.

In most cases, we found the cys-Mms6 could not be sufficiently photodegraded during exposure to laser light in the interferometer and a complete layer of MNPs was formed on the surface. When using a high exposure dose of 100 J cm^–2^ we occasionally saw some evidence of patterning during SEM analysis (such as the images displayed in Fig. 5). However, patterning was not achieved with the same level of consistency as when the OEG-thiolate SAM was patterned and the cys-Mms6 was used as a backfill (as shown in Fig. 2 and 3). This is simply because photodegrading the protein is much more difficult, and requires much more energy than the simpler OEG-thiolate.

Clearly, there is scope to improve the process described here to generate nanoscale dot patterns of Mms6 or other biomineralising proteins, something that we are currently exploring. Attention may also need to be given to the choice of magnetic material, as we have previously shown that the soft magnetic properties of magnetite (*i.e.* its low coercivity) mean that may not suitable for use in magnetic data storage.^22–24^ Techniques such as biopanning have uncovered many novel peptide sequences which can interact with more technologically relevant nanomaterials that are not found in nature.^47^ Furthermore, we have recently shown that enhanced biopanning can achieve morphological reproduction^48^ using protein biopanning.^49^ Some of these biopanning procedures are able to biotemplate the formation of MNPs of Pt alloys of Co and Fe, and organise these materials onto surfaces.^11,12,50^ These materials, when in the L1_0_ phase, are considered ideal for BPM, as their high magnetocrystalline energy means they maintain their magnetic domain at dimensions of a few nanometres.^51–53^

## Conclusions

We have developed a combined top-down and bottom-up strategy for successfully producing nanoscale patterns of magnetite MNPs. This is the first time IL has been used in combination with MNP biomineralisation to create such functional nanopatterned magnetic surfaces. IL was shown to produce distinct patterns, and the Mms6 protein patterned areas successfully biotemplate uniform MNPs under mild reaction conditions. However, this study is only a first step towards the production of BPM, but there are many new areas for the future development of this methodology. We are currently working to produce dot arrays that would be more geometrically appropriate for BPM, and reduce the pattern size even further. In addition, this work represents a powerful proof-of-concept for future adaptation to produce a range of different nanomaterials on different nanopatterned surfaces, from alternative MNPs to other functional materials such as quantum dots. This could be used to create a vast array of novel nanotechnology, from BPM to lab-on-a-chip sensing devices, potentially transforming nanotechnology fabrication.
