# Supplementary material for: A novel design strategy for nanoparticles on nanopatterns: interferometric lithographic patterning of Mms6 biotemplated magnetic nanoparticles
Source: J Mater Chem C Mater. 2016 Jan 12;4(18):3948–55. doi: 10.1039/c5tc03895b (PMC4894075; doi:10.1039/c5tc03895b)
Supplement: Supplementary file 1 [file TC-004-C5TC03895B-s001.pdf]

Electronic Supplementary Material (ESI) for Journal of Materials Chemistry C.
This journal is © The Royal Society of Chemistry 2016

A novel design strategy for nanoparticles on nanopatterns: interferometric lithographic patterning of Mms6 biotemplated magnetic nanoparticles

S. M. Bird,[a] O. El-Zubir,[a,b] A. E. Rawlings,[a] G. J. Leggett[a] and S. S. Staniland[a*]

[a] Department of Chemistry, The University of Sheffield, Sheffield, S3 7HF, UK.
[b] Chemical Nanoscience Laboratories, School of Chemistry, University of Newcastle, Newcastle upon Tyne, NE1 7RU, UK.

Supplementary information.

Mms6 protein

Amino acid sequence: MGSHHHHHHHGSTENLYFQGCPRMGGTIWTGKG LGLGLGLGLGAWGPIILGVVGAGAVYAYMKSRDIESAQSDEEVELRDALA

C : Cysteine
H : Histidine purification tag
N : Tobacco etch virus (TEV) cleavage site
G : Wild type Mms6

Number of amino acids: 87

Molecular weight: 8.92 kDa

pI: 6.2

Synthesis: Produced in the Staniland Group following the method described in Bird *et al.*[1]

Supplementary Fig. 1S Key information on the cysteine-tagged Mms6 (cys-Mms6) protein.

[1] S. M. Bird, J. M. Galloway, A. E. Rawlings, J. P. Bramble and S. S. Staniland, Nanoscale, 2015, 7, 7340 - 7351.

Friction force microscopy (FFM)

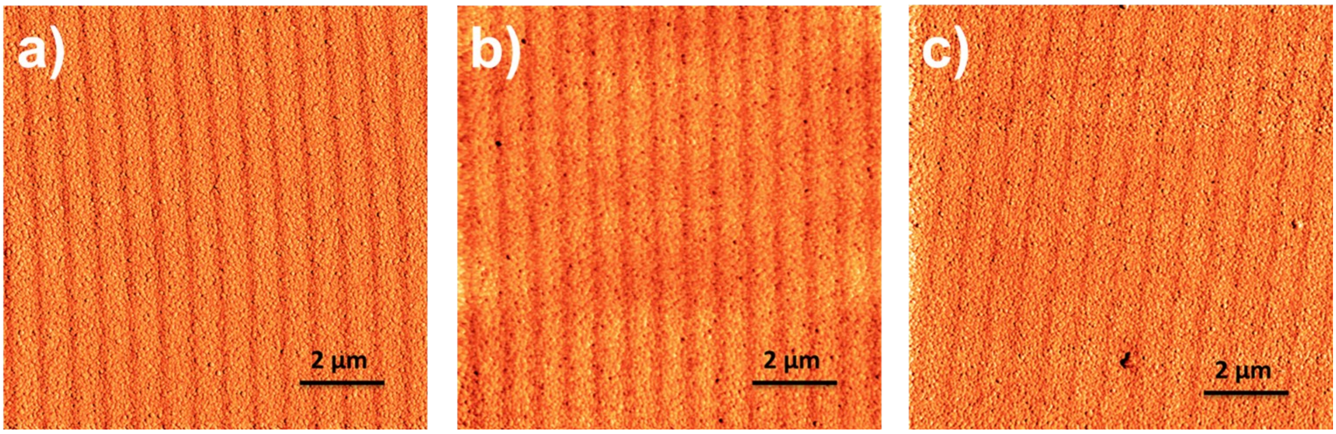

Supplementary Fig. 2S Friction force microscopy (FFM) images of a mixed SAM of PEG and a COOH terminated thiol formed on gold that was patterned by IL exposure at a dose of 20 J cm^{-3} (a), 30 J cm^{-3} (b) and 40 J cm^{-3} (c) and backfilled with a CH_3 terminated thiol SAM to provide contrast in FFM.
